# The ESC Cardio-Oncology Guidelines: A Roadmap for Clinical Practice and Generating Needed Evidence

**DOI:** 10.1016/j.jaccao.2022.10.010

**Published:** 2022-12-06

**Authors:** Joseph A. Sparano, Gagan Sahni

**Affiliations:** a Division of Hematology and Medical Oncology, Tisch Cancer Institute, Department of Medicine, Icahn School of Medicine at Mount Sinai, New York, New York, USA; b Division of Cardiology, Department of Medicine, Icahn School of Medicine at Mount Sinai, New York, New York, USA

**Keywords:** cancer survivorship, cardiomyopathy, echocardiography, heart failure

The 2022 European of Society of Cardiology (ESC) Guidelines on cardio-oncology, developed by the ESC Cardio-Oncology Task Force in collaboration with the European Hematology Association, the European Society for Therapeutic Radiology and Oncology, and the International Cardio-Oncology Society, represent an essential resource for health care providers, including not only cardiologists, cardio-oncologists, and primary care physicians, but also medical oncologists prescribing cancer therapeutics that may adversely impact cardiovascular health, and ultimately cancer survivorship.^1^ It is estimated that there will be 26 million cancer survivors by 2040, the vast majority of whom will be at high risk for cardiovascular disease due to older age, a higher prevalence of risk factors common to both cancer and cardiovascular disease, and the increasing recognition of both short-term and long-term cardiovascular effects of cancer therapeutics.^2^ The ESC guidelines provide a roadmap for cardiovascular risk assessment, cardiac monitoring, and recommendations for primary and secondary interventions to mitigate cardiovascular complications from potentially cardiotoxic cancer therapies, many of which may be curative of the cancer. The guidelines encompass the entire spectrum of contemporary cancer therapeutics associated with cardiac effects, ranging from cytotoxic agents such as anthracyclines and anti-human epidermal growth factor receptor 2 (HER2)–directed therapies to newer therapies targeting immune checkpoints, Bruton’s tyrosine kinase, vascular endothelial growth factor, and the proteasome, as well as other targets and modalities. Some therapies produce short-term cardiotoxic effects unique to the specific drug class (eg, Bruton’s tyrosine kinase inhibitors and arrhythmias; immune checkpoint blockade and myocarditis; anthracyclines and heart failure), whereas others may contribute to increased atherosclerotic risk years later (eg, endocrine therapies for breast and prostate cancer).

Although the 2022 ESC guidelines are uniquely comprehensive in scope and represent a major overhaul of prior ESC guidelines published in 2016,^3^ there is no shortage of similar guidelines dating back to as early as 2014, including those developed by the American Society of Echocardiography and European Association of Cardiovascular Imaging,^4^ the Canadian Cardiovascular Society,^5^ the American Society of Clinical Oncology (ASCO),^6^ and the European Society of Medical Oncology.^7^ Discrepancy between some of these guidelines, and the lack of evidence supporting many of them, led the French Working Group of Cardio-Oncology to analyze and harmonize the most recent American and European guidelines into a “pragmatic approach” for clinical implementation.^8^ For medical oncologists and cardio-oncologists at the forefront of breast cancer patient care, availability of the newest 2022 ESC guidelines raise the question of how they may be similar to or differ from prior guidelines, whether they should be followed in totality or selectively, whether adherence to some or all of these guidelines will or should be applied as quality measures for cancer care, and the level of evidence supporting key recommendations.

In order to exemplify the conundrum posed by having discordant expert-derived guidelines, we focus here on discordance between the 2022 ESC guidelines and 2016 ASCO guidelines for monitoring of patients receiving anti-HER2 therapy, as summarized in Table 1, and also highlight the level of evidence supporting them. Although both guidelines advise cardiovascular risk stratification based on the cardiovascular risk associated with the specific anticancer therapy to be used and the individual’s baseline cardiovascular disease history and risk factors, the ESC guidelines recommend surveillance with 2-dimensional transthoracic echocardiography (TTE) at baseline and every 3 months during anti-HER2 therapy in all patients irrespective of risk, whereas the prior ASCO guidelines recommend surveillance only in high-risk individuals, and at a frequency determined by the treating physician based on clinical judgment and patient circumstances. There is also a new recommendation unique to the ESC guidelines for the use of blood cardiac troponins and natriuretic peptides for patients who have had prior anthracycline therapy, which adds another layer of monitoring and cost.

**Table 1:** Comparison of 2016 ASCO and 2022 ESC Guidelines for Monitoring Patients Receiving Anti-HER2 Therapy

	ASCO Guidelines	ESC Guidelines
Electrocardiogram	Baseline	Baseline
2-dimensional transthoracic echocardiography	Low risk: • Baseline High risk: • Baseline • Frequency after be determined by health care professional based on clinical judgment and patient circumstances	Low risk (recommended): • Baseline • 3, 6, 9, and 12 mo, and 12 mo after therapy completion (may reduce to every 4 mo if 3-mo study normal) High risk (recommended): • Baseline • 3, 6, 9, and 12 mo, and 12 mo after therapy completion
Blood cardiac troponin and natriuretic peptide	Not recommended	Low risk (may be considered): • Baseline • 3, 6, 9, and 12 mo, and 3 and 12 mo after therapy completion^a^ High risk (recommended at baseline, should be considered thereafter): • Baseline • 3, 6, 9, and 12 mo, and 3 and 12 mo after therapy completion^a^

ESC guidelines refer to cardiovascular toxicity monitoring in patients receiving neoadjuvant or adjuvant anti-HER2–targeted therapies for nonmetastatic disease or first year in metastatic disease.

ASCO = American Society of Clinical Oncology; ESC = European Society of Cardiology; HER2 = human epidermal growth factor receptor 2.

a Measurement of natriuretic peptide and/or cardiac troponin is recommended in all patients with cancer if these biomarkers are going to be used during treatment monitoring. Baseline cardiac troponin measurement should be considered in low- and moderate-risk patients after anthracycline chemotherapy but prior to starting anti-HER2–targeted therapies for cardiovascular toxicity risk prediction.

The primary value of cardiac surveillance, whether with TTE and/or cardiac biomarkers, is to not only to confirm symptomatic cardiac therapy–related cardiac dysfunction (CTRCD), the term used by the ESC panel, but perhaps more importantly detect asymptomatic CTRCD that may trigger interrupting or even withholding anti-HER2 therapy, and/or initiating cardioprotective therapy such as beta blockers, angiotensin-converting enzyme inhibitors, or angiotensin receptor blockers. According to Table 25 of the ESC guidelines, anti-HER2 therapy should be interrupted if there is symptomatic moderate-to-severe CTRCD (as defined in Table 3), or asymptomatic CTCRD with left ventricular ejection fraction <40%. In addition, the guidelines recommend that anti-HER2 therapy be continued with guideline-directed medical therapy for asymptomatic CTRCD with a left ventricular ejection fraction of at least 40%. Although these criteria provide clear and valuable guidance regarding continuing anti-HER2 therapies in those with asymptomatic CTRCD, there remain 3 fundamental issues regarding universal screening in all cardiac risk groups and normal cardiac function at baseline for asymptomatic CTRCD.

First, the risk of CTRCD associated with anti-HER2 therapy, specifically monoclonal antibodies including trastuzumab or the trastuzumab/pertuzumab combination, is greatest when following anthracycline therapy, which are much less commonly used as adjuvant or neoadjuvant therapy for breast cancer given the comparable effectiveness of nonanthracycline regimens. For patients with a low baseline cardiovascular risk receiving nonanthracycline chemotherapy regimens, the likelihood in asymptomatic patients meeting any of the criteria for interrupting anti-HER2 therapy and/or initiating cardioprotective therapy by the 2022 ESC guidelines is very low for both localized and metastatic disease,^9^^,^^10^ although precise estimation is problematic due to lack of consistency in definitions of CTRCD in individual reports.

Second, other commonly used HER2-targeted therapies such as anti-HER2 tyrosine kinase inhibitors (eg, tucatinib) and antibody-drug conjugates (eg, trastuzumab emtansine and trastuzumab deruxtecan) are also associated with very low CTRCD risk.^10^ Some anti-HER2 therapies (eg, trastuzumab, pertuzumab, trastuzumab emtansine, tyrosine kinase inhibitors) are approved for early and advanced stage breast cancer that overexpresses HER2 protein (typically associated with HER2 gene amplification), whereas others (eg, trastuzumab deruxtecan) have also shown benefit in patients with advanced breast cancer and other cancers with low levels of HER2 protein expression (and not associated with HER2 gene amplification). The ESC guidelines for monitoring patients receiving anit-HER2 therapy will therefore have broad implications for cardiac surveillance for cancer therapy. It is critical that we select the right monitoring plan for the right anti-HER2 agent and the right patient using a risk-based model integrating the CTCRD risk associated with the specific anti-HER2 agent and the underlying cardiovascular risk of the individual patient—one size does not fit all.

Finally, the level of evidence supporting the specific ESC recommendations for cardiac monitoring during and in the first 12 months after HER2 therapy is low. For each guideline, the ESC panel provided a class of recommendation (Class 1 = recommended; Class 2a = should be considered; Class 2b = may be considered; Class 3 = not recommended), and level of evidence supporting the guideline, including Level of Evidence: A (data derived from multiple randomized clinical trials or meta-analyses), Level of Evidence: B (data derived from a single randomized clinical trial or large nonrandomized studies), or Level of Evidence: C (consensus of opinion of the experts and/or small studies, retrospective studies, registries). Of the 9 recommendations applicable to cardiac surveillance in patients receiving anti-HER2 therapy, 7 are supported by consensus opinion and the lowest level of evidence (C), whereas a lower B level of evidence was cited as supporting 3 monthly cardiac surveillance using TTE. In fact, of the 156 Class 1 recommendations summarized at the end of the ESC guidelines, only 5 (3%) were supported by the highest level of evidence (Level of Evidence: A), whereas 118 (76%) were supported by expert consensus without robust data (Level of Evidence: C). This highlights the need to harvest and mine real-world data, develop prospective registries designed to address knowledge gaps, and develop traditional and pragmatic clinical trials, all of which will ultimately generate the evidence required to better inform and revise current guidelines. The ESC panel indeed recognized “gaps in the evidence”—we agree that substantial gaps exist and suggest that evaluation and validation of cardiac surveillance algorithms for the entire spectrum of anti-HER2 therapy be added as a knowledge gap and identified as a research priority. We also particularly emphasize the evidence gap and need for additional research regarding “optimal follow-up…after treatment for cancer,” especially after cardiotoxic anthracycline therapy, in which follow-up surveillance beyond 12 months after completing therapy is currently not recommended in the ESC guidelines, but risk of insidious and delayed CTRCD may be high.

In conclusion, the 2022 ESC Guidelines on cardio-oncology represent an impressive and important effort for providing greater clarity in defining cardiotoxicities of emerging anticancer agents, minimizing the potential cardiovascular complications of myriad cancer therapies, and assuring healthy cancer survivorship. The authors of the guidelines and the organizations supporting their development need to be congratulated for their important efforts and encouraged to lead the charge in generating the evidence required to support and refine the current guidelines.

## Funding Support and Author Disclosures

This work was supported in part by grants from the Department of Health and Human Services and the National Institutes of Health, National Cancer Institute (P30CA196521). Dr Sparano has served as a paid consultant for AstraZeneca, Roche, and Seagen. Dr. Sahni has reported that she has no relationships relevant to the contents of this paper to disclose.
